# Supplementary material for: 3D imaging of colorectal cancer organoids identifies responses to Tankyrase inhibitors
Source: PLoS One. 2020 Aug 18;15(8):e0235319. doi: 10.1371/journal.pone.0235319 (PMC7433887; doi:10.1371/journal.pone.0235319)
Supplement: S1 Checklist — (DOCX) [file pone.0235319.s001.docx]

**ARRIVE GUIDELINES CHECKLIST**

**INTRODUCTION**

Background

3D tumour organoids provide a platform in which complex cell-cell interactions can be studied. However, complex 3D models provide a challenging platform for the quantitative analysis of drug responses of therapies that have differential effects on tumour cell subpopulations. Tumour organoids were generated from colorectal cancer patients and were tested for their responses to inhibitors of Tankyrase (TNKSi) which are known to modulate Wnt signalling. Using compounds with 3 orders of magnitude difference in cellular mechanistic potency together with image-based assays, morphometric analyses were shown to capture subtle alterations in organoid responses to TNKSi inhibitors that are consistent with activity against a cancer stem cell subpopulation.

Objectives

The presence of cancer stem cells is often identified by transplantation of tumour cells within immune-deficient animal models and subsequent formation of tumours. To assess whether both functional and phenotypic effects of TNKSi within a Wnt-dependent, TNKSi-sensitive colorectal organoids would translate to a reduction in tumour growth *in vivo*, TNKSi-exposed and control organoids were grafted subcutaneously into immune-deficient mice.

**METHODS**

Ethical Statement

The work described here was carried out under the conditions of the UK Home Office project licence PPL 30/3279.

Study Design

1. *Cohorts:* TNKSi-exposed organoid xenografts (16 mice) and control organoid xenografts (16 mice). This was a take experiment; the times elapsed from xenografting to appearance of palpable tumours (>5 mm) were the output data.
2. *Mouse allocation to cohorts:* on the basis of cage; the cages were stocked by animal technicians.
3. *Experimental Unit:* Single mouse.

Experimental Procedures

1. *Organoid Culture Conditions:* Organoids were cultured in a growth medium of Advanced DMEM/F12 supplemented with penicillin/streptomycin, 2mM GlutaMAX, 10 mM HEPES,1 X B27 supplement, 1 X N2 supplement (all Invitrogen), 1 mM N-acetyl-L-cysteine (Sigma-Aldrich), 100 ng/ml mouse recombinant Noggin (Peprotech), 10% RSpo-1 conditioned medium, 40% Wnt-3A conditioned medium, 500 nM A83-01 (Tocris) and 10 μM SB202190 (Sigma).
2. *Preparation of Organoids for Xenografts:* One day before xenografting, the organoids were trypsinised to single cells during passaging and seeded at 1,000 cells/50 μl Matrigel blob. 15 blobs were used per treatment. The organoids were exposed to 2 μM MSC2524070 TNKSi inhibitor or vehicle (0.0002% DMSO). On the day of xenograft, the organoids were resuspended in 50% Matrigel/50% growth medium and transferred to the mouse facility on ice.
3. *Xenograft procedure:* Mice were kept in sterilised, filter top cages. Xenografting of mice were carried out in a Class II flow hood with HEPA filtered air in a procedure room. The skin over the right flank of each mouse was “tented” and 100 ml organoid suspension was injected subcutaneously using a syringe with a 26-gauge needle. A separate needle was used for each mouse. The mice were monitored over 30 minutes before return to the holding room.
4. *Monitoring:* Mice were monitored every two days for appearance of palpable tumours. Tumours were measured using a caliper to determine if they were >5 mm diameter. Mice with tumours >5 mm diameter were euthanased by cervical dislocation.

Experimental Animals

1. *Animals Used:* Male NOD/Scid/γ-irradiated (NSG) mice of 35-42 days of age. They weighed 20-25g.
2. *Source of Animals:* Charles River
3. *Genotype:* NOD.CB17-Prkdc^scid^/NCrCrl

Housing and Husbandry

1. *Cages:* Large, sterilised filter top cages with sterile litter. There were 5-6 mice per cage. Cages were changed once a week by animal technicians in a Class II flow hood with HEPA filtered air.
2. *Conditions:* 25°C, 12 hours light/12 hours dark
3. *Welfare Assessments:*
   1. **Prior to experiment:** transfer passport
   2. **During:** Every 2 days
   3. **After:** Mice were euthanised on appearance of a palpable tumour (>5 mm)

Sample Size

1. *Specify the total number of animals used in each experiment, and the number of animals in each experimental group:* 16 animals per cohort x 2 cohorts = 32 animals total.
2. *Sample size calculation:* Power calculation
3. *Number of experiments:* 1

Allocating Animals to Experimental Groups

1. *Allocation:* on the basis of cage
2. *Order of Treatment:* all mice were xenografted with either control or TNKSi-exposed organoids on the same day

Experimental Outcomes

The appearance of tumours and the times elapsed from xenografting to appearance of palpable tumours (>5 mm) were the output data and these were plotted on a Kaplan-Meier graph.

Statistical Methods

The two Kaplan-Meier plots were compared statistically using the Log Rank (Mantel-Cox) test and the P value was 0.0322.

**RESULTS**

Baseline Data

Mice weighed 20-25g and the weight did not alter during the experiment.

Numbers Analysed

There were 16 animals in each treatment group (n=2 treatment groups; 32 animals in total). All animals in the experiment (32/32) developed tumours.

Outcomes and Estimation

All mice developed tumours from the organoid implants but they appeared on different time periods after xenografting. The times elapsed from xenografting to appearance of palpable tumours (>5 mm) were plotted on a Kaplan-Meier graph.

Adverse Effects

There were no adverse effects.

**DISCUSSION**

Interpretation/Scientific implications

1. *Interpretation:* Exposure of these Wnt-dependent colorectal organoids to tankyrase inhibitors significantly delayed the appearance of palpable (>5 mm) tumours.
2. *Study Limitations:* The grafting was sub-cutaneous, not orthotopic.
3. *Implications on 3R (Refinement, Replacement, Reduction Objectives):* Use of organoid source material (ODX) for xenografting instead of PDX material will reduce the number of mice required.

Generalisability/Translation

The use of tumour organoids will potentially revolutionise the pre-clinical testing of novel therapeutic compounds. Organoids are fully representative, three-dimensional miniature versions of the patient tumour tissue from which they are derived. Moreover, several studies have shown that responses of organoids in vitro to standard of care drugs correlates with responses to the same drugs of the tumour from which the organoids were derived. In comparison, the use of cell lines in drug discovery are thought to have contributed to the failure of several drug-discovery programs in early-stage clinical trials.

Funding

Innovate UK grant 9776 and Cancer Research UK Programme Grant C1295/A15937H
