# Supplementary material for: 3D imaging of colorectal cancer organoids identifies responses to Tankyrase inhibitors
Source: PLoS One. 2020 Aug 18;15(8):e0235319. doi: 10.1371/journal.pone.0235319 (PMC7433887; doi:10.1371/journal.pone.0235319)

Supplementary Figure S1

Hematoxylin & Eosin (H & E) staining of FFPE tissue sections derived from primary colorectal tumour patient material, with organoid counterparts. Scale bar = 100 µm.


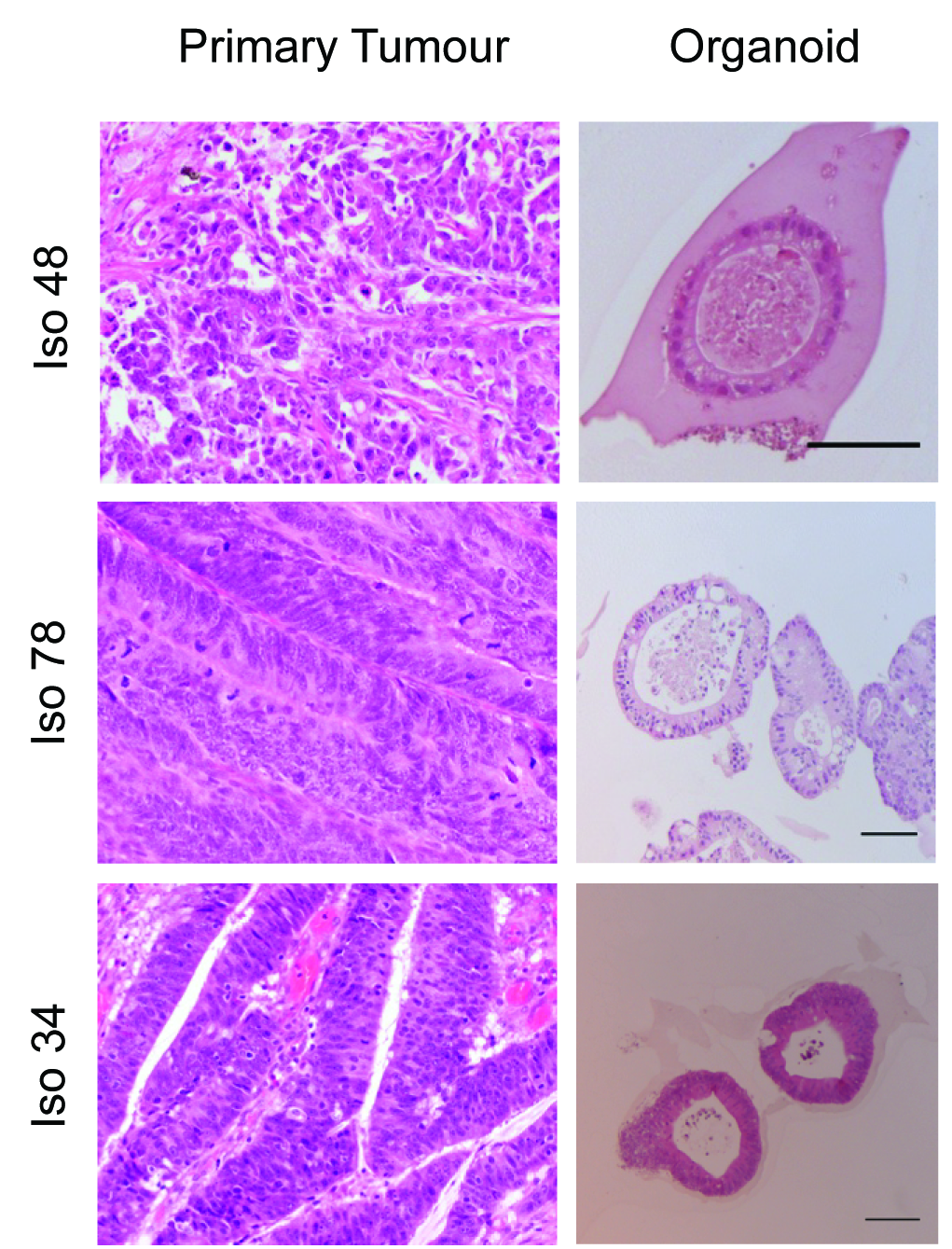

Supplement: S1 Fig — Hematoxylin & Eosin (H & E) staining of FFPE tissue sections derived from primary colorectal tumour patient material, with organoid counterparts. Scale bar = 100 μm. (DOCX) [file pone.0235319.s002.docx]
