# Supplementary material for: 3D imaging of colorectal cancer organoids identifies responses to Tankyrase inhibitors
Source: PLoS One. 2020 Aug 18;15(8):e0235319. doi: 10.1371/journal.pone.0235319 (PMC7433887; doi:10.1371/journal.pone.0235319)

Supplementary Figure S2

Full scans of immunoblots. Related to Figure 2B. Organoids and cell line controls were treated with TNKSi C1 (organoids 15 nM, cell line 50nM) and blotted for TNKS1/2 top or Axin2 bottom . Novex Sharp pre-stained protein standards (Invitrogen; LC5800) were loaded as per manufacturers instructions.


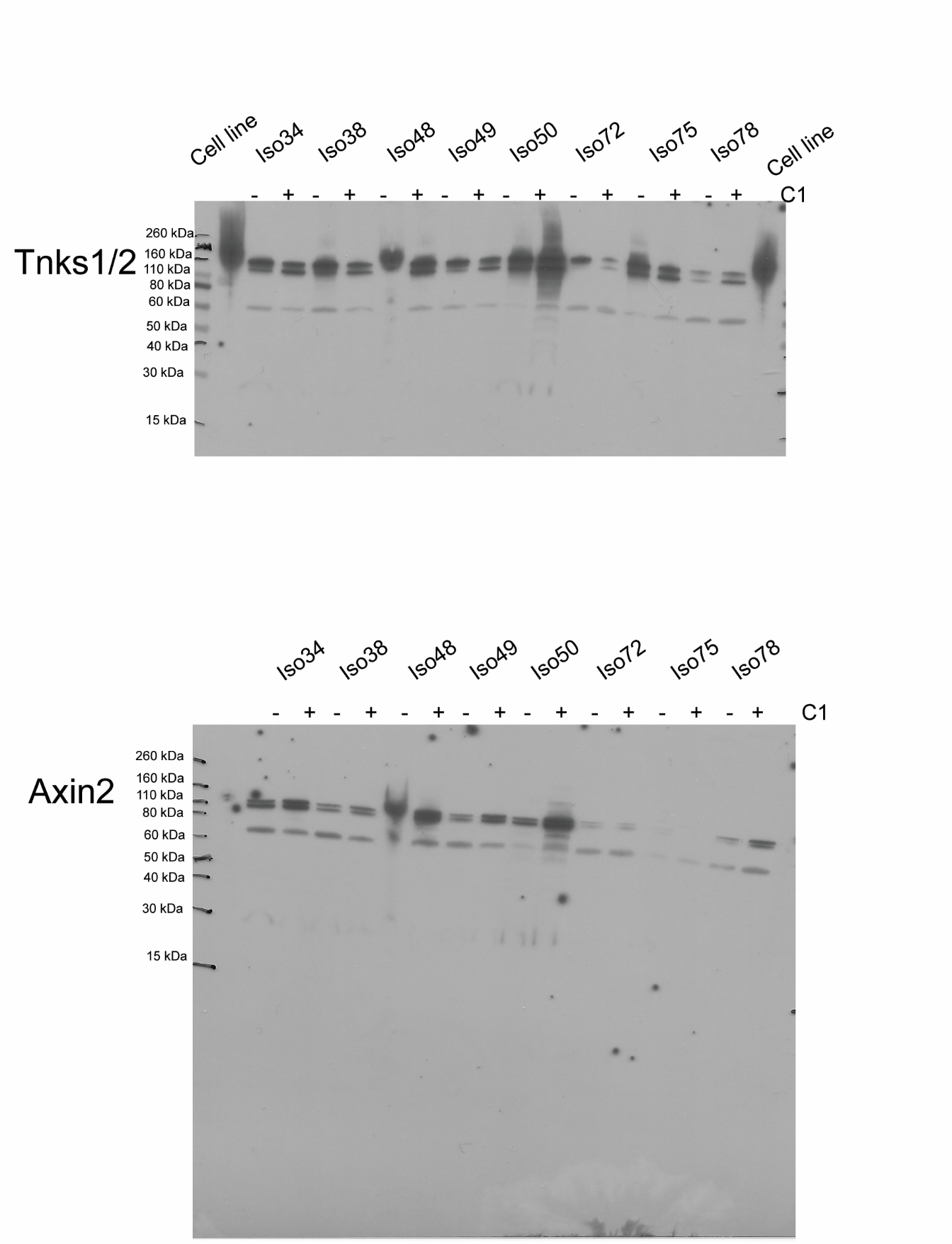

Supplement: S2 Fig — Full scans of immunoblots. Related to Fig 2B. Organoids and cell line controls were treated with TNKSi C1 (organoids 15 nM, cell line 50nM) and blotted for TNKS1/2 top or Axin2 bottom. Novex Sharp pre-stained protein standards (Invitrogen; LC5800) were loaded as per manufacturers instructions. (DOCX) [file pone.0235319.s003.docx]
