# Supplementary material for: 3D imaging of colorectal cancer organoids identifies responses to Tankyrase inhibitors
Source: PLoS One. 2020 Aug 18;15(8):e0235319. doi: 10.1371/journal.pone.0235319 (PMC7433887; doi:10.1371/journal.pone.0235319)
Supplement: S3 Fig — (A) Representative images of healthy intestinal organoids treated with C1 (12.5 nM, 100 nM) or DMSO (scale bar = 100 μm). (B) Dose-response curves of organoids treated with C1 for six days. Organoids previously established in suitable culture conditions were passaged by mechanical trituration into small (<100 μm) structures, overlaid with media to allow recovery for 24 hours, then treated with a dose range of C1 (0.31 nM– 100 nM) or DMSO control (0.1% in media). Organoids were treated for a total of 6 days with metabolic activity determined using Cell Titer Glo 3D, with readings normalized to DMSO control (100%), n = 3 independent experiments, with results shown as mean ± S.D. (DOCX) [file pone.0235319.s004.docx]

Supplementary Figure S3

**A.** Representative images of healthy intestinal organoids treated with C1 (12.5 nM, 100 nM) or DMSO (scale bar = 100 µm) **B.** Dose-response curves of organoids treated with C1 for six days. Organoids previously established in suitable culture conditions were passaged by mechanical trituration into small ( <100 µm) structures, overlaid with media to allow recovery for 24 hours, then treated with a dose range of C1 ( 0.31 nM – 100 nM) or DMSO control (0.1% in media). Organoids were treated for a total of 6 days with metabolic activity determined using Cell Titer Glo 3D, with readings normalized to DMSO control (100%), n=3 independent experiments, with results shown as mean ± S.D.

**
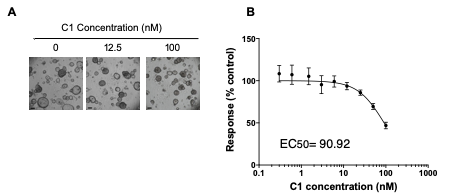
**
