# Supplementary material for: 3D imaging of colorectal cancer organoids identifies responses to Tankyrase inhibitors
Source: PLoS One. 2020 Aug 18;15(8):e0235319. doi: 10.1371/journal.pone.0235319 (PMC7433887; doi:10.1371/journal.pone.0235319)

Supplementary Figure S4

Representative images of organoids exposed to low and high dose TNKSi (C1, C2 and C3 for 6 days) after image analysis. Projections of the Hoechst (Blue) and Phalloidin-rhodamine (Red) signal are overlaid with the cell and lumen mask (Green). The images show 10% of the original image. Scale bar = 500 µm.


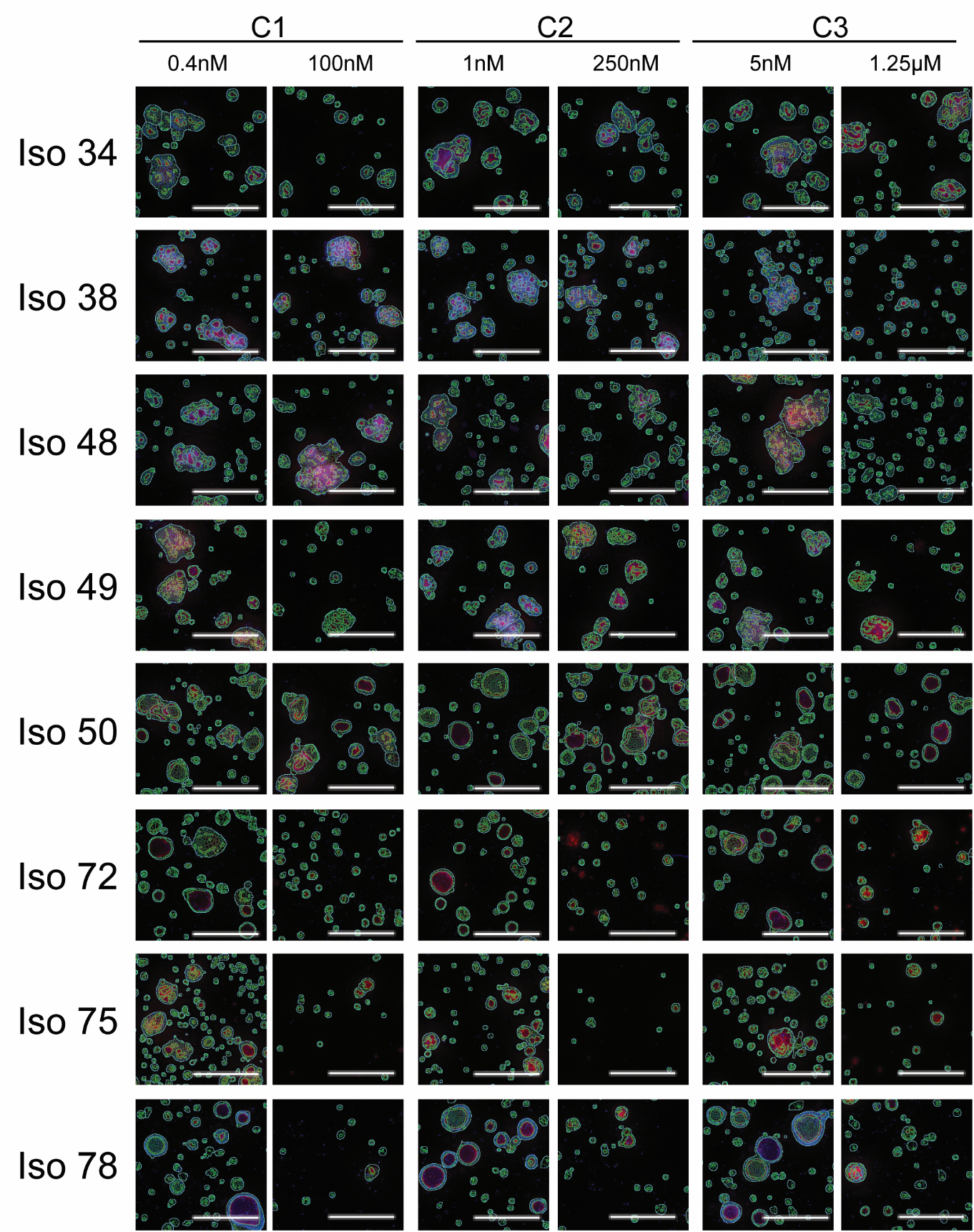

Supplement: S4 Fig — Representative images of organoids exposed to low and high dose TNKSi (C1, C2 and C3 for 6 days) after image analysis. Projections of the Hoechst (Blue) and Phalloidin-rhodamine (Red) signal are overlaid with the cell and lumen mask (Green). The images show 10% of the original image. Scale bar = 500 μm. (DOCX) [file pone.0235319.s005.docx]
