# Supplementary material for: 3D imaging of colorectal cancer organoids identifies responses to Tankyrase inhibitors
Source: PLoS One. 2020 Aug 18;15(8):e0235319. doi: 10.1371/journal.pone.0235319 (PMC7433887; doi:10.1371/journal.pone.0235319)

Supplementary Figure S5

Representative images of organoids (Iso 50, Iso 72 and Iso 75) exposed to high dose TNKSi (C1; 100 nM , C2; 250 nM and C3; 1.25µM, 6 days) versus DMSO control after image analysis. Z-Projections of the Hoechst (Blue) and Phalloidin-rhodamine (Red) signal are overlaid with the organoid and lumen mask (yellow and green). Individual z-section masks illustrate changes in morphology of organoids in treatment versus DMSO conditions. The images show 10% of the original image. Scale bar = 500 µm.


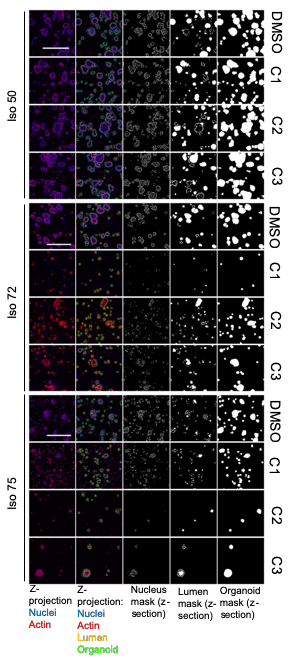

Supplement: S5 Fig — Representative images of organoids (Iso 50, Iso 72 and Iso 75) exposed to high dose TNKSi (C1; 100 nM, C2; 250 nM and C3; 1.25μM, 6 days) versus DMSO control after image analysis. Z-Projections of the Hoechst (Blue) and Phalloidin-rhodamine (Red) signal are overlaid with the organoid and lumen mask (yellow and green). Individual z-section masks illustrate changes in morphology of organoids in treatment versus DMSO conditions. The images show 10% of the original image. Scale bar = 500 μm. (DOCX) [file pone.0235319.s006.docx]
