# Supplementary material for: 3D imaging of colorectal cancer organoids identifies responses to Tankyrase inhibitors
Source: PLoS One. 2020 Aug 18;15(8):e0235319. doi: 10.1371/journal.pone.0235319 (PMC7433887; doi:10.1371/journal.pone.0235319)
Supplement: S6 Fig — A Representative confocal images of organoids stained with an Lgr5 and Cytokeratin 20 antibody following six days of exposure to C1 (15 nM) or control (DMSO, 0.1%). Sensitive organoids demonstrated an overall reduction in the number of Lgr5 positive cells and increase in Cytokeratin 20 positive cells following treatment. (DOCX) [file pone.0235319.s007.docx]

Supplementary Figure S6

**A**. Representative confocal images of organoids stained with an Lgr5 and Cytokeratin 20 antibody following six days of exposure to C1 (15 nM) or control (DMSO, 0.1%). Sensitive organoids demonstrated an overall reduction in the number of Lgr5 positive cells and increase in Cytokeratin 20 positive cells following treatment.
