# Supplementary material for: 3D imaging of colorectal cancer organoids identifies responses to Tankyrase inhibitors
Source: PLoS One. 2020 Aug 18;15(8):e0235319. doi: 10.1371/journal.pone.0235319 (PMC7433887; doi:10.1371/journal.pone.0235319)
Supplement: S1 Table — (DOCX) [file pone.0235319.s008.docx]

# Supplementary Table S1.

Primary antibodies used for fluorescence microscopy.

| **Primary Antibody** | **Source** | **Catalogue Number** | **Dilution** | **IgG** |
| --- | --- | --- | --- | --- |
| Ki67 | Millipore | AB9260 | 1:100 | Rabbit |
| β-catenin | BD | 610154 | 1:250 | Mouse |
| Lgr5 | BD | 562733 | 1:250 | Rat |
| Cytokeratin 20 (KRT20) | Abcam | AB76126 | 1:100 | Rabbit |
|  |  |  |  |  |
