# Supplementary material for: 3D imaging of colorectal cancer organoids identifies responses to Tankyrase inhibitors
Source: PLoS One. 2020 Aug 18;15(8):e0235319. doi: 10.1371/journal.pone.0235319 (PMC7433887; doi:10.1371/journal.pone.0235319)
Supplement: S2 Table — (DOCX) [file pone.0235319.s009.docx]

# Supplementary Table S2.

Primers used for qRT-PCR by SYBR green.

| **Gene** | **Forward Sequence** | **Reverse Sequence** |
| --- | --- | --- |
| AXIN1 | CTGGATACCTGCCGACCTTA | CCGGCATTGACATAATAGGG |
| AXIN2 | GCGATCCTGTTAATCCTTATCAC | AATTCCATCTACACTGCTGTC |
| TNKS1 | CCGCGTGTCTGTTGTAGAGT | ACAGAAGCCCCATGCCTTAC |
| TNKS2 | TGGTGTGGGAGCCAAGTCTA | GTGGCAATTCACTCCTCTTCA |
| LGR5 | GAGTTACGTCTTGCGGGAAAC | TGGGTACGTGTCTTAGCTGATTA |
| ASCL2 | TGACCTGGGGCGTAATAAAG | ACACAGGCTTCTCCCTAGCA |
| KRT20 | ACGCCAGAACAACGAATACC | ACGACCTTGCCATCCACTAC |
| DKK1 | CCCAGGCTCTGCAGTCAGCG | CGCACGGGTACGGCTGGTAG |
| GAPDH | TGAAGGTCGGAGTCAACGGA | CCATTGATGACAAGCTTCCCG |
