# Supplementary material for: 3D imaging of colorectal cancer organoids identifies responses to Tankyrase inhibitors
Source: PLoS One. 2020 Aug 18;15(8):e0235319. doi: 10.1371/journal.pone.0235319 (PMC7433887; doi:10.1371/journal.pone.0235319)
Supplement: S1 Appendix — The mutations reported in the table are those found to have been previously described as likely oncogenic and curated within the COSMIC and cBioportal databases. (DOCX) [file pone.0235319.s010.docx]

# Supplementary Appendix S1

Whole Exome sequencing analysis for each organoid line. The mutations reported in the table are those found to have been previously described as likely oncogenic and curated within the COSMIC and cBioportal databases.
